# Diabetes Is Associated With Rapid Progression of Aortic Stenosis: A Single-Center Retrospective Cohort Study

**DOI:** 10.3389/fcvm.2021.812692

**Published:** 2022-02-23

**Authors:** Kangning Han, Dongmei Shi, Lixia Yang, Meng Xie, Rongrong Zhong, Zhijian Wang, Fei Gao, Xiaoteng Ma, Yujie Zhou

**Affiliations:** ^1^Beijing Anzhen Hospital, Capital Medical University, Beijing, China; ^2^Beijing Institute of Heart, Lung and Blood Vessel Disease, Beijing, China; ^3^The Key Laboratory of Remodeling-Related Cardiovascular Disease, Ministry of Education, Beijing, China; ^4^Department of Echocardiogram, Beijing Institute of Heart, Lung, and Blood Vessel Diseases, Beijing Anzhen Hospital, Capital Medical University, Beijing, China; ^5^Faculty of Medicine, Imperial College London, London, United Kingdom

**Keywords:** diabetes, aortic stenosis, rapid progression, transthoracic echocardiography, valvular disease

## Abstract

**Background:**

Mounting evidence indicates that rapid progression of aortic stenosis (AS) is significantly associated with poor prognosis. Whether diabetes accelerates the progression of AS remains controversial.

**Objectives:**

The purpose of the present study was to investigate whether diabetes was associated with rapid progression of AS.

**Methods:**

We retrospectively analyzed 276 AS patients who underwent transthoracic echocardiography at least twice with a maximum interval ≥ 180 days from January 2016 to June 2021. AS severity was defined by specific threshold values for peak aortic jet velocity (V_max_) and/or mean pressure gradient. An increase of V_max_ ≥ 0.3 m/s/year was defined as rapid progression. The binary Logistic regression models were used to determine the association between diabetes and rapid progression of AS.

**Results:**

At a median echocardiographic follow-up interval of 614 days, the annual increase of V_max_ was 0.16 (0.00–0.41) m/s. Compared with those without rapid progression, patients with rapid progression were older and more likely to have diabetes (*P* = 0.040 and *P* = 0.010, respectively). In the univariate binary Logistic regression analysis, diabetes was associated with rapid progression of AS (OR = 2.02, *P* = 0.011). This association remained significant in the multivariate analysis based on model 2 and model 3 (OR = 1.93, *P* = 0.018; OR = 1.93, *P* = 0.022). After propensity score-matching according to V_max_, diabetes was also associated rapid progression of AS (OR = 2.57, *P* = 0.045).

**Conclusions:**

Diabetes was strongly and independently associated with rapid progression of AS.

## Background

Aortic stenosis (AS) is one of the most common valvular heart diseases affecting up to nearly 10% of the senior population (1–3). AS is a progressive disease and its rapid progression has been shown to be associated with poor prognosis (4–8). Patients with rapid progression of AS may not be easily identified at the initial diagnosis; however, these patients should be considered intervention early rather than at the onset of symptoms (8). Indeed, one study showed that early intervention in asymptomatic severe AS patients with rapid progression of AS was associated with a significant reduction in mortality (9). Many studies defined rapid progression of AS as an increase of aortic jet velocity (V_max_) ≥ 0.3 m/s/year (7, 10). Both ACC/AHA and ESC guidelines consider an increase of V_max_ ≥ 0.3 m/s/year as one of the indications for AS intervention (classes of recommendations: IIa, level of evidence: B-C) (11, 12). Although the pathogenesis of AS is similar to that of coronary artery disease (CAD), the determinants of its rapid progression remain unclear (13). Although many promising pharmacotherapeutic studies have shown the potential benefits in detaining the progression of AS, currently, there is no effective drug treatment for AS, and valve replacement represents the only treatment for end-stage AS (14). Identification of risk factors for rapid progression of AS may allow for its secondary prevention.

Two studies have shown that diabetes can accelerate the progression of AS (15, 16). However, one of the studies only included mild AS patients above the age of 60 and defined AS progression by the yearly increase of peak transvalvular gradient (15). The other study comprised more than 99% of male patients and defined AS progression according to the annual decrease of aortic valve area (16). Whether diabetes can significantly accelerate AS progression as shown by V_max_ is unknown. Since the rapid increase in V_max_ is currently an indication for AS intervention, therefore, the present study was to investigate the association of diabetes with the progression of AS according to V_max_.

## Methods

### Patient Population

**Figure 1:**
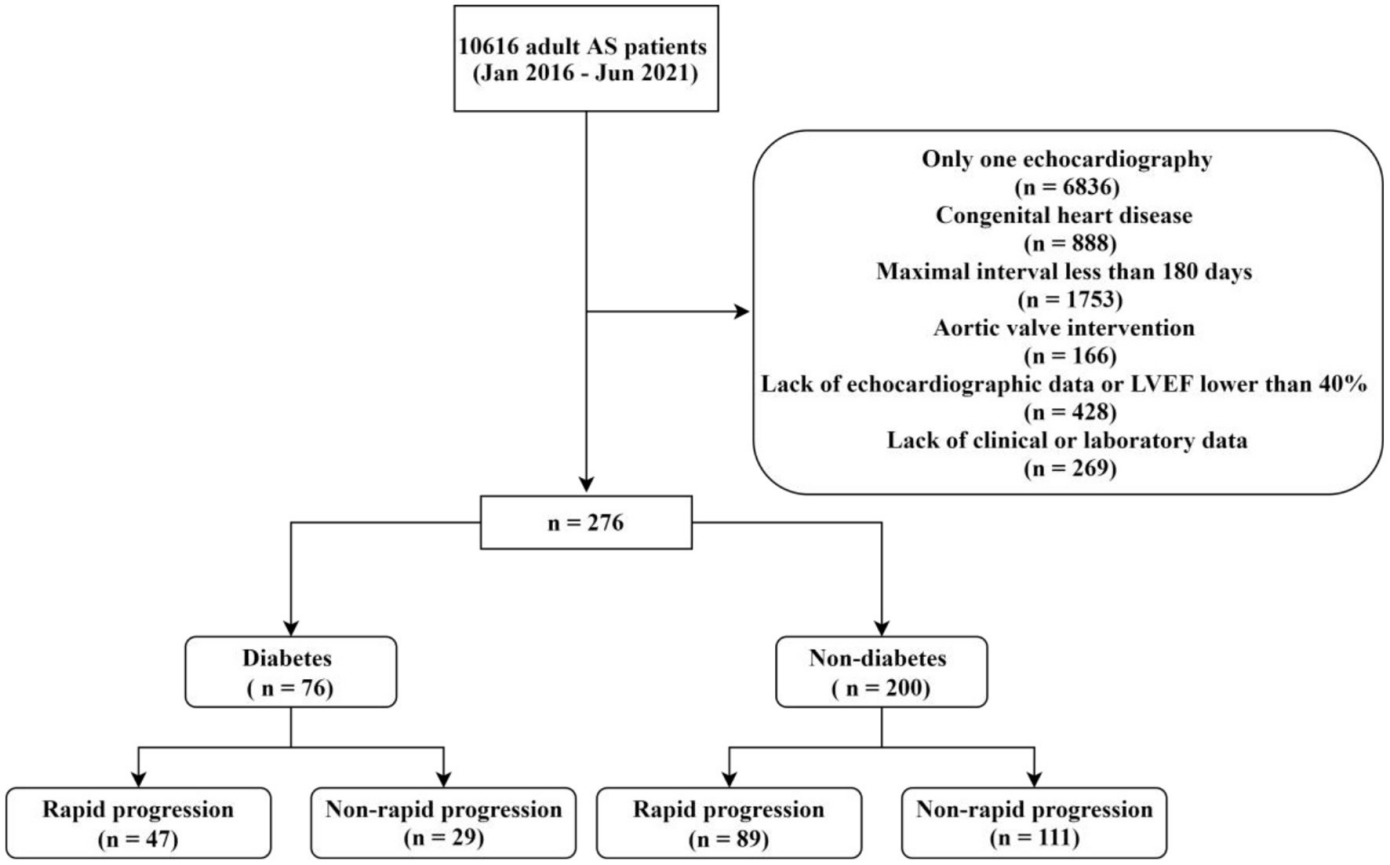
Flow chart of the study.

Patients diagnosed with AS were retrospectively identified by the electronic medical system of Beijing Anzhen Hospital, Capital Medical University from January 2016 to June 2021. AS was diagnosed by transthoracic echocardiography according to V_max_ and/or mean pressure gradient (MPG). All patients underwent comprehensive echocardiography by experienced sonographers and the following diagnostic criteria were used: (1) mild AS (V_max_ 2.00–2.99 m/s and/or MPG 10.0–19.9 mmHg); (2) moderate AS (V_max_ 3.00–3.99 m/s and/or MPG 20.0–39.9 mmHg); (3) severe AS (V_max_ ≥ 4.00 m/s and/or MPG ≥ 40.0 mmHg). Then, a total of 3,780 adult patients who underwent transthoracic echocardiography at least twice were selected. If the patient had more than one echocardiography after the first echocardiography, the one with the highest V_max_ was selected as the last echocardiography. The exclusion criteria were: (1) congenital heart disease other than bicuspid aortic valve (BAV); (2) history of aortic valve surgery or transcatheter aortic valve implantation; (3) lack of clinical history, echocardiographic or laboratory data; (4) maximal interval from the first echocardiography to the last echocardiography < 180 days; (5) left ventricular ejection fraction (LVEF) at the first or last echocardiography < 40% (Figure 1). Patients with a history of atrial fibrillation (AF) or concomitant significant valvular diseases were not excluded. For patients with AF, echocardiography was performed over at least five cardiac cycles, and the V_max_ was averaged. The first (baseline) and the last echocardiography were used for analysis. Information about age, sex, smoking, drinking, hypertension, diabetes, dyslipidemia, CAD, heart failure, chronic kidney disease (CKD), BAV, and medications (such as statins, ACEI/ARBs, and β-blockers) during the observation period were obtained from the electronic medical system or telephone interviews. Rapid progression of AS was defined as the rate of V_max_ progression ≥ 0.3 m/s/year.

### Statistical Analysis

Continuous variables with normal distribution were expressed as mean ± standard deviation, otherwise median and quartile. For continuous variables, the unpaired *t*-test and Mann-Whitney *U*-test were used as appropriate. Categorical variables were expressed as numbers and percentages, where the χ^2^ test or Fisher exact test was used accordingly. Three different Logistic regression models were used to determine the association of diabetes with rapid progression: (1) unadjusted; (2) adjusted for sex and age; (3) adjusted for sex, age, hypertension, CKD, dyslipidemia, and smoking. Paired patient data depending on the diagnosis of diabetes was acquired by propensity score-matching based on the baseline V_max_. All statistical analyses were performed by SPSS 26.0 (SPSS, Inc, Chicago, Illinois) and a 2-sided *P*-value < 0.05 was considered statistically significant.

## Results

A total of 276 AS patients constituted the study population, including 69 (25.0%) mild, 129 (46.7%) moderate, and 78 (28.3%) severe AS. The baseline characteristics of the population were summarized in Table 1. Among the study population, the mean age was 65 ± 11 years, 131 (47.5%) were female, 76 (27.5%) had diabetes, and 82 (29.7%) had BAV. Patients with rapid progression were older (*P* = 0.040) and more likely to have diabetes (*P* = 0.010). Compared with those without diabetes, patients with diabetes had lower baseline V_max_ and MPG, but had higher rates of hypertension, dyslipidemia, CAD, and use of medications (Table 2). The progression of AS in diabetic patients was more pronounced than in non-diabetic patients (Table 2).

**Table 1:** Baseline characteristics of the study population according to V_max_ progression.

**Variables**	**All patients** **(*n* = 276)**	**Non-rapid progression** **(*n* = 140)**	**Rapid progression** **(*n* = 136)**	***P*-value**
Age (years)	65 ± 11	64 ± 12	66 ± 9	0.040
Female, *n* (%)	131 (47.5)	69 (49.3)	62 (45.6)	0.539
Smoking, *n* (%)	68 (24.6)	33 (23.6)	35 (25.7)	0.677
Hypertension, *n* (%)	198 (71.7)	101 (72.1)	97 (71.3)	0.880
Diabetes, *n* (%)	76 (27.5)	29 (20.7)	47 (34.6)	0.010
FPG (mmol/L)	6.58 ± 2.25	6.31 ± 2.05	6.85 ± 2.41	0.051
Dyslipidemia, *n* (%)	163 (59.1)	78 (55.7)	85 (62.5)	0.252
TG (mmol/L)	1.43 ± 0.99	1.45 ± 1.04	1.40 ± 0.93	0.648
TC (mmol/L)	4.42 ± 1.31	4.48 ± 1.39	4.36 ± 1.23	0.448
LDL-C (mmol/L)	2.63 ± 1.12	2.65 ± 1.21	2.60 ± 1.02	0.666
HDL-C (mmol/L)	1.25 ± 0.36	1.28 ± 0.37	1.22 ± 0.35	0.184
CAD, *n* (%)	123 (44.6)	59 (42.1)	64 (47.1)	0.411
CKD, *n* (%)	27 (9.8)	15 (10.7)	12 (8.8)	0.597
Heart failure, *n* (%)	20 (7.2)	11 (7.9)	9 (6.6)	0.691
Statins, *n* (%)	140 (50.7)	67 (47.9)	73 (53.7)	0.334
ACEI/ARBs, *n* (%)	91 (33.0)	41 (29.3)	50 (36.8)	0.186
β-blockers, *n* (%)	122 (44.2)	61 (43.6)	61 (44.9)	0.830
Baseline LVEF (%)	65 (60–68)	65 (60–68)	65 (59–68)	0.826
Baseline V_max_ (m/s)	3.52 ± 0.78	3.58 ± 0.84	3.45 ± 0.71	0.185
Baseline MPG (mmHg)	29.82 ± 15.40	30.68 ± 16.66	28.94 ± 13.18	0.338
BAV, *n* (%)	82 (29.7)	47 (33.6)	35 (25.7)	0.154

*FPG, fasting plasma glucose; TG, triglycerides; TC, total cholesterol; LDL-C, low-density lipoprotein-cholesterol; HDL-C, high-density lipoprotein-cholesterol; CAD, coronary artery disease; CKD, chronic kidney disease; ACEI/ARBs, angiotensin converting enzyme inhibitor/angiotensin receptor blockers; LVEF, left ventricular ejection fraction; MPG, mean pressure gradient; BAV, bicuspid aortic valve*.

**Table 2:** Baseline characteristics of the study population according to the with and without diabetes.

**Variables**	**All patients** **(*n* = 276)**	**Without diabetes** **(*n* = 200)**	**With diabetes** **(*n* = 76)**	***P*-value**
Age (years)	65 ± 11	64 ± 11	66 ± 9	0.156
Female, *n* (%)	131 (47.5)	99 (49.5)	32 (42.1)	0.272
Smoking, *n* (%)	68 (24.6)	48 (24.0)	20 (26.3)	0.690
Hypertension, *n* (%)	198 (71.7)	134 (67.0)	64 (84.2)	0.005
Dyslipidemia, *n* (%)	163 (59.1)	105 (52.5)	58 (76.3)	<0.001
TG (mmol/L)	1.43 ± 0.99	1.37 ± 0.88	1.58 ± 1.22	0.125
TC (mmol/L)	4.42 ± 1.31	4.48 ± 1.38	4.29 ± 1.14	0.282
LDL-C (mmol/L)	2.63 ± 1.12	2.69 ± 1.18	2.47 ± 0.95	0.167
HDL-C (mmol/L)	1.25 ± 0.36	1.26 ± 0.35	1.23 ± 0.38	0.401
CAD, *n* (%)	123 (44.6)	78 (39.0)	45 (59.2)	0.003
CKD, *n* (%)	27 (9.8)	21 (10.5)	6 (7.9)	0.515
Heart failure, *n* (%)	20 (7.2)	12 (6.0)	8 (10.5)	0.195
Statins, *n* (%)	140 (50.7)	88 (44.0)	52 (68.4)	<0.001
ACEI/ARBs, *n* (%)	91 (33.0)	54 (27.0)	37 (48.7)	0.001
β-blockers, *n* (%)	122 (44.2)	78 (39.0)	44 (57.9)	0.005
Baseline LVEF (%)	65 (60–68)	65 (59–68)	65 (60–68)	0.471
Baseline V_max_ (m/s)	3.52 ± 0.78	3.58 ± 0.80	3.34 ± 0.72	0.023
Baseline MPG (mmHg)	29.82 ± 15.40	31.10 ± 15.73	26.71 ± 13.05	0.033
BAV, *n* (%)	82 (29.7)	64 (32.0)	18 (23.7)	0.177
Rapid progression, *n* (%)	136 (49.3)	89 (44.5)	47 (61.8)	0.010

*Abbreviations as in Table 1*.

The median time interval from the first echocardiography to the last echocardiography was 614 days (interquartile range: 350–932 days). During the period, the V_max_ and MPG were increasing. The V_max_ increased from 3.52 ± 0.78 m/s to 3.88 ± 0.87 m/s, with an annual increase of 0.16 (0.00–0.41) m/s. The MPG was increased from 30 ± 15 mmHg to 36 ± 18 mmHg, with an annual increase of 2.78 (0.00–6.86) mmHg. Compared with those without diabetes, patients with diabetes had a more significant V_max_ progression [0.21 (0.09–0.41) m/s/year vs. 0.13 (−0.02 to 0.42) m/s/year, *P* = 0.024].

**Figure 2:**
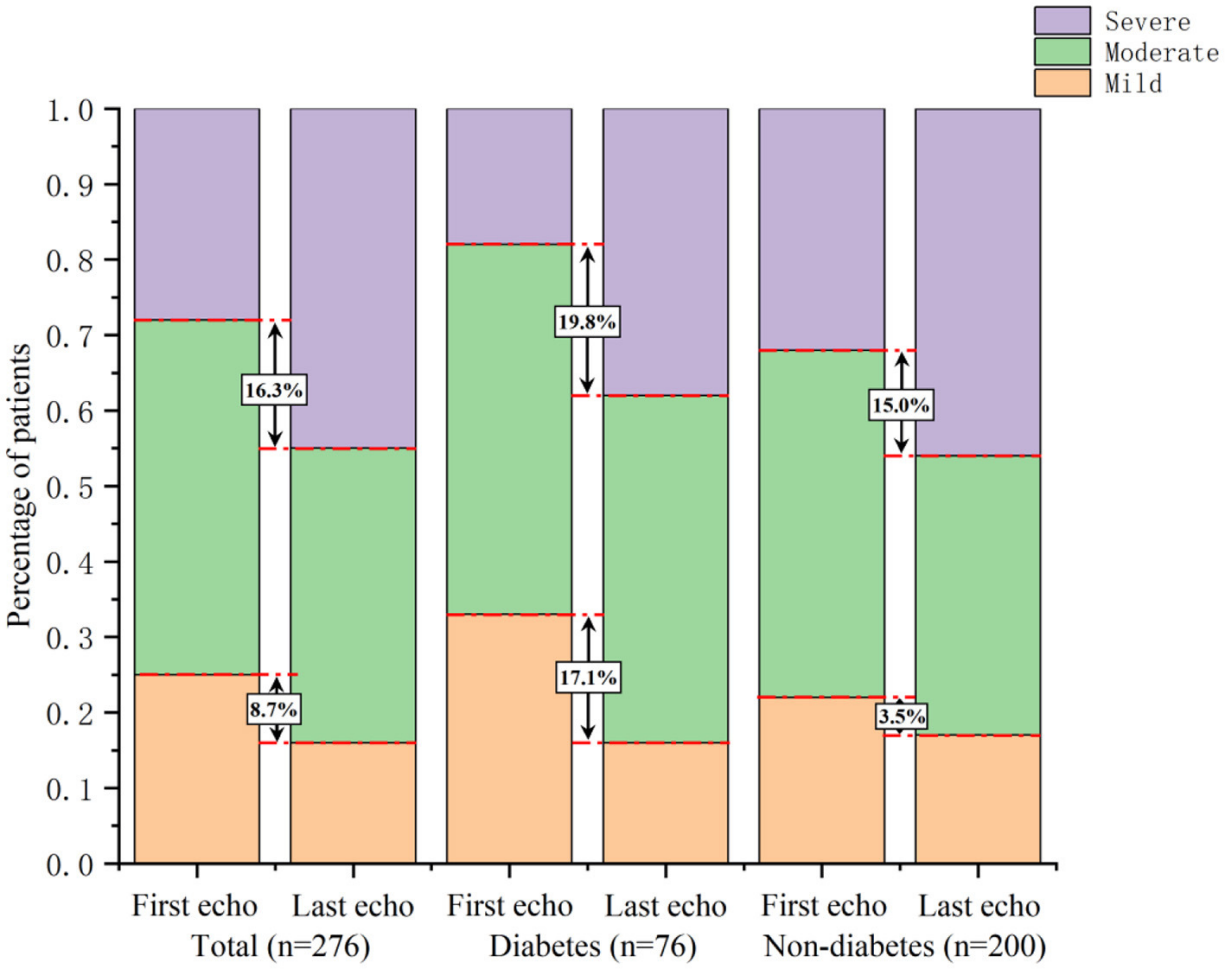
Comparison of AS severity changes between patients with and without diabetes. In diabetic patients, the proportion of moderate-to-severe AS increased by 17.1%, and severe AS increased by 19.8%. In non-diabetic patients, the proportion of moderate-to-severe AS increased by 3.5%, and severe AS increased by 15.0%.

At follow-up, the proportion of patients with severe AS increased by 16.3%, while the proportions of patients with mild and moderate AS decreased by 8.7% and 7.6%, respectively. In the 76 diabetic patients, the proportion of moderate-to-severe AS increased by 17.1%, and particularly, severe AS increased by 19.8%. In the patients without diabetes, the proportion of moderate-to-severe AS increased by 3.5%, and severe AS increased by 15.0% (Figure 2).

In the univariate binary Logistic regression analysis (model 1), diabetes was associated with rapid progression of AS (unadjusted OR = 2.02, 95% CI: 1.18–3.47, *P* = 0.011). After adjustment for potential confounders in model 2 and model 3, this association remained significant (Table 3). After propensity score-matching, multivariate Logistic regression analysis (adjusted by sex, age, hypertension, CKD, dyslipidemia, and smoking) showed diabetes was associated with rapid progression of AS (OR = 2.57, 95% CI: 1.02–6.47, *P* = 0.045).

**Table 3:** Odds ratios and 95% confidence intervals for rapid progression according to with and without diabetes.

	**With vs. without diabetes**	
**Logistic models**	**OR (95% CI)**	***P*-value**
Model 1	2.02 (1.18–3.47)	0.011
Model 2	1.93 (1.12–3.33)	0.018
Model 3	1.93 (1.10–3.39)	0.022

*Model 1: Unadjusted*.

*Model 2: Adjusted for sex, age*.

*Model 3: Adjusted for sex, age, hypertension, CKD, dyslipidemia, smoking*.

## Discussion

In this study, we demonstrated that diabetes was significantly and independently associated with the rapid progression of AS. Previous study of Deutscher et al. showed that diabetes was associated with the development of AS (17). Likewise, diabetes was significantly associated with an increased incidence of AS, according to a recent retrospective cohort study involving 78,805 patients with diabetes and 78,805 patients without diabetes (18). Another retrospective cohort study comprised 71,813 subjects showed that diabetes was associated with a 34% increased risk of AS (19). Diabetes leads to elevated valvular and plasma accumulation of glycation end products in AS patients. Additionally, there was a positive correlation between the amount of glycation end products and the severity of AS in diabetic patients (20).

Diabetes can lead to aortic valve calcification, which is a major cause of AS, especially in the elderly. The study of Katz et al. revealed that diabetes was associated with an increased risk of aortic valve calcification detected by computed tomography (21). Furthermore, diabetes has been shown to be associated with stenotic and non-stenotic aortic valve calcification (22). Tucureanu et al. found increased expression of cell adhesion molecules, extracellular matrix remodeling, and osteogenic markers in hyperlipidemia ApoE^−/−^ diabetic mice (23). High glucose can induce osteogenic molecules and increase calcium deposits by increasing the expression of cytokines, cell adhesion molecules, and matrix metalloproteinases in valvular endothelial cells and interstitial cells (24). In the animal model, diabetes can lead to early aortic valve mineralization and calcific AS (25).

Diabetes can also cause inflammation and degeneration that can lead to AS. AS is associated with macrophages, mast cells, T cells and other immune cells infiltrations, emphasizing the role of local inflammation in AS (26–28). In severe AS patients before aortic valve replacement, diabetic individuals had higher levels of C-reactive protein in plasma and aortic valve (29). Diabetes can decrease the number and induce dysfunction of endothelial progenitor cells, impairing valve endothelial repair (30). Indeed, diabetes has been shown to be associated with aortic valve and bioprosthetic aortic valve degeneration (31, 32). At the molecular level, diabetes can lead to the upregulation of biglycan, which may contribute to aortic valve degeneration (33).

In AS patients, diabetes was associated with a worse prognosis. It has been reported that diabetes can impair coronary microvascular function in patients with asymptomatic AS (34). Some studies have shown that diabetes has an adverse effect on hypertrophic remodeling and is associated with reduced systolic function (35, 36). Moreover, diabetes was demonstrated to be associated with systemic inflammatory response syndrome after aortic valve replacement (37). A previous study by Lancellotti et al. showed that diabetes was associated with higher mortality in severe AS patients (38).

In this study, we found diabetes could accelerate the progression of AS, and particularly, lead to a rapid progression (V_max_ ≥ 0.3 m/s/year). Several studies have demonstrated the association between diabetes and AS progression. The study of Aronow et al. first showed that diabetes was associated with the progression of AS. However, this study only included mild AS patients aged more than 60 years and defined AS progression according to annual decrease of peak transvalvular gradient (15). The study of Kamalesh et al. showed that diabetes significantly accelerated the progression of AS in patients with moderate AS. However, this study included more than 99% of male patients and defined AS progression according to annual decrease of aortic valve area (16). Diabetes was found to be a strong risk factor for the development of severe AS in a prospective cohort study which included 1.12 million individuals without valvular disease history (39). Moreover, diabetes has been shown to accelerate the progression of AS by enhancing the inflammatory response measured by C-reactive protein, where an increase in the inflammatory response was observed within the aortic valve of AS patients (29). Additionally, the density of immune cell infiltration within aortic valve was demonstrated to be correlated with the progression of AS (40). Of note, an increased inflammatory response has also been shown to be associated with the development and progression of diabetes, underlying a potential link between diabetes and the progression of AS (41).

## Limitation

Several limitations must be considered when interpreting the results of our study. First, this study was a single-center retrospective study, so the influence of unmeasured and residual confounding variables could not be excluded. Second, the data of this study were limited to the Chinese population, so the ethnic difference cannot be eradicated. Third, this study did not perform the external validation, which may hamper the general applicability of the current findings. Fourth, aortic valve area was not included in this study since it was not measured routinely by sonographers in our cardiovascular center. Fifth, to better reflect the impact of diabetes on the progression of AS in the real world, similar to many previous studies, we did not exclude patients with AF and concomitant significant valve diseases. Excluding patients with those conditions may determine the association between diabetes and AS progression more directly and accurately. Future studies on such patients are needed.

## Conclusions

In this study, we demonstrated that diabetes was strongly and independently associated with the rapid progression of AS. Further prospective studies are needed to confirm our results.

## Data Availability Statement

The raw data supporting the conclusions of this article will be made available by the authors, without undue reservation.

## Ethics Statement

The studies involving human participants were reviewed and approved by Beijing Anzhen Hospital, Capital Medical University. Written informed consent for participation was not required for this study in accordance with the national legislation and the institutional requirements.

## Author Contributions

KH and XM analyzed the data and drafted the manuscript. KH and MX extracted the echocardiography data from the electronic medical system. MX guided the echocardiography analysis. RZ analyzed the data and drew the figures. XM and YZ designed the study and revised the manuscript. All authors contributed to the article and approved the submitted version.

## Funding

This work was supported by National Key Research and Development Program of China (2017YFC0908800); China Postdoctoral Science Foundation (2021M692253); Beijing Postdoctoral Research Foundation (2021-ZZ-023); Beijing Municipal Administration of Hospitals Mission Plan (SML20180601).

## Conflict of Interest

The authors declare that the research was conducted in the absence of any commercial or financial relationships that could be construed as a potential conflict of interest.

## Publisher's Note

All claims expressed in this article are solely those of the authors and do not necessarily represent those of their affiliated organizations, or those of the publisher, the editors and the reviewers. Any product that may be evaluated in this article, or claim that may be made by its manufacturer, is not guaranteed or endorsed by the publisher.
